# Association of preoperative prognostic nutritional index with risk of postoperative delirium: A systematic review and meta-analysis

**DOI:** 10.3389/fmed.2022.1017000

**Published:** 2023-01-09

**Authors:** Kuo-Chuan Hung, Chong-Chi Chiu, Chih-Wei Hsu, Chun-Ning Ho, Ching-Chung Ko, I-Wen Chen, Cheuk-Kwan Sun

**Affiliations:** ^1^Department of Anesthesiology, Chi Mei Medical Center, Tainan City, Taiwan; ^2^Department of Hospital and Health Care Administration, College of Recreation and Health Management, Chia Nan University of Pharmacy and Science, Tainan City, Taiwan; ^3^Department of General Surgery, E-Da Cancer Hospital, Kaohsiung City, Taiwan; ^4^School of Medicine, College of Medicine, I-Shou University, Kaohsiung City, Taiwan; ^5^Department of Medical Education and Research, E-Da Cancer Hospital, I-Shou University, Kaohsiung City, Taiwan; ^6^Department of Psychiatry, Kaohsiung Chang Gung Memorial Hospital and Chang Gung University College of Medicine, Kaohsiung City, Taiwan; ^7^Department of Medical Imaging, Chi Mei Medical Center, Tainan City, Taiwan; ^8^Department of Health and Nutrition, Chia Nan University of Pharmacy and Science, Tainan City, Taiwan; ^9^Institute of Biomedical Sciences, National Sun Yat-sen University, Kaohsiung City, Taiwan; ^10^Department of Anesthesiology, Chi Mei Medical Center, Liouying, Tainan City, Taiwan; ^11^Department of Emergency Medicine, E-Da Hospital, I-Shou University, Kaohsiung City, Taiwan

**Keywords:** postoperative delirium, prognostic nutritional index, general anesthesia, surgery, nutrition

## Abstract

**Study objective:**

To assess the association between prognostic nutritional index (PNI) and risk of postoperative delirium (POD) in adult patients.

**Methods:**

MEDLINE, Google scholar, EMBASE, and Cochrane library databases were searched from inception till April 2022. The primary outcome was the association between PNI and the risk of POD, while the secondary outcomes were correlations of other prognostic factors with POD risk. The correlation between PNI and the incidence of POD was assessed with three approaches: Difference in preoperative PNI between POD and non-POD groups (Model 1) as well as the association of PNI as a continuous parameter (Model 2) or as a binary variable (i.e., low vs. high using a PNI cut-off value of 50) (Model 3) with POD risk.

**Results:**

Analysis of nine observational studies published from 2010 to 2021 recruiting 3,743 patients showed a POD incidence of 6.4–35%. Our meta-analysis demonstrated a lower PNI among patients in the POD group (MD: −3.78, 95% CI: −4.85 to −2.71, *p* < 0.0001, *I*^2^ = 54.2%) compared to the non-POD group (Model 1). Pooled results revealed a negative association between PNI and POD risk for both Model 2 (OR: 0.91, 95% CI: 0.86–0.97, *p* = 0.002, *I*^2^ = 71%) and Model 3 (OR: 1.68, 95% CI: 1.26–2.23, *p* < 0.0001, *I*^2^ = 0%). Besides, while our results supported an age-dependent increase in POD risk, other factors including body-mass index, surgical time, health status, hypertension, diabetes mellitus, and male gender were non-significant predictors of POD.

**Conclusion:**

Our results demonstrated a negative association between PNI and POD, which warrant further large-scale studies for validation.

**Systematic review registration:**

https://www.crd.york.ac.uk/prospero/, identifier CRD42022323809.

## 1. Introduction

It has been expected that the world population of people aged over 65 years would triple from 461 million in 2004 to as high as two billion by 2050 (1). An expected increase in the number of older surgical patients (2) means a corresponding elevation in the incidence of postoperative complications. Postoperative delirium (POD), which occurs in 17–61% of patients after major surgical procedures (3–5), mostly affects the aged population and is characterized by fluctuating disturbances of consciousness, perception, cognition, and attention (6, 7). Previous studies have shown that not only could POD increase the risks of institutionalization and dementia as well as decline in activities of daily living but it could also contribute to mortality (8–11). The importance of primary prevention of POD (12, 13) is demonstrated by the finding that POD is preventable in up to 30–40% of patients before its onset (14) as well as the ineffectiveness of treatment efforts for decreasing the duration, severity, or likelihood of recurrence following an initial episode of delirium (12, 13, 15). Therefore, identification of patients at high risk of POD combined with early interventions could minimize the incidence of POD and improve postoperative outcomes (12).

Previous studies reported malnutrition as a poor prognostic factor independently associated with surgical complication and 1-year mortality (16–18). The prognostic nutritional index (PNI), an indicator of nutritional status, was first proposed by Buzby in 1980 (19) and modified by Onodera et al. who summarized its calculation in a formula: PNI = albumin (g/L) + 5 × absolute lymphocyte count (10^9^/L) to quantitatively estimate the operative risk in patients with cancer (20). Previous applications of PNI were mainly focused on prognosis prediction in cancer patients because of their poor nutritional status characterized by a suppression of albumin concentration and absolute lymphocyte count (20–24). Several studies have also suggested that PNI could be used as a tool for predicting the occurrence of POD in older patients (25–29). Nevertheless, the effectiveness of the use of PNI as a predictor for POD has not been addressed in a systematic approach.

This meta-analysis aimed at exploring the association of preoperative PNI with POD in adult patients receiving non-cardiac surgery under general anesthesia. Other risk factors such as hypertension or surgical time were also investigated.

## 2. Materials and methods

The present study complied with the PRISMA (Preferred Reporting Items for Systematic Reviews and Meta-Analyses) guidelines when conducting the current meta-analysis (PROSPERO CRD42022323809), in which the selection of eligible studies, collection of relevant data, and assessment of the risks of bias were independently performed by two authors. Disagreements were resolved through discussion.

### 2.1. Data sources and searches

The databases of MEDLINE, Google scholar, the Cochrane Library, and EMBASE were searched from inception to April 7, 2022 by utilizing a combination of keywords and MeSH terms. Supplementary Table 1 summarizes the procedures of literature search using Medline as an example. We imposed no restriction on year of publication, language, and sample size. To ensure completeness of our literature search, the lists of references of the retrieved articles and the published meta-analyses were scrutinized to identify possibly eligible studies.

### 2.2. Inclusion and exclusion criteria

Eligibility of a study was based on the following criteria: (a) Studies assessing the association of preoperative PNI with the incidence of POD in adult patients receiving surgery under general anesthesia, and (b) those providing sufficient detail for computation or extraction of data on individual odds ratio (OR) and 95% confidence intervals (CIs). Observational (i.e., prospective and retrospective) studies and randomized controlled trials were eligible for inclusion. The exclusion criteria included: (1) studies focusing on pediatric population, (2) articles presented as letters, reviews, case reports, conference abstracts, or other forms of publication instead of original investigation, (3) those evaluating postoperative cognitive dysfunction, and (4) those using unvalidated diagnostic criteria for POD.

### 2.3. Data extraction

The following data were collected from all studies: first author’s name, year of publication, characteristics of patients (e.g., gender distribution), number of study patients, body mass index (BMI), health status according to the American Society of Anesthesiologists’ Physical Status Classification (ASA-PS), comorbidities (e.g., hypertension), type and duration of surgical procedures, PNI values, incidence of POD, and country. For each study, we obtained the OR together with the 95% CIs through matched or adjusted analyses. We recorded the adjusted OR for a study that gave both unadjusted and adjusted OR. If a continuous variable was dichotomized, we computed the OR using the number of cases and controls based on the cut-off value of PNI defined in that study. We contacted the authors of studies in which the necessary data were missing or additional clarification was needed.

### 2.4. Definitions and outcomes

The primary outcome was the correlation between PNI and POD risk, while the secondary outcomes were the associations of other prognostic factors (e.g., BMI) with POD risk. The definition of POD was based on well-established diagnostic criteria in each study. To reduce the impact of confounding factors, we conducted subgroup analyses on the types of surgery to evaluate the association of PNI with the risk of POD.

### 2.5. Assessment of risks of bias for the included studies

Two independent reviewers assessed the risk of bias for individual study in accordance with the six domains described in the Quality in Prognostic Studies (QUIPS) tool (30). The risk of a study was assigned as low, moderate, or high for each domain. Accordingly, we considered the overall risk of bias of a study to be low when all or most domains of that study were rated as low (or low to moderate) (31).

### 2.6. Data synthesis and analysis

We used the comprehensive Meta−Analysis (CMA) V3 software (Biostat, Englewood, NJ, USA) for all statistical analyses. The overall effect size was computed with the reported raw data of event counts for primary analysis. Considering the inclusion of observational studies in this study, a random-effects model was adopted to generate an overall OR to serve as the main summary measure of effect size as previously reported (32, 33). Statistical heterogeneity of effect size was assessed with *I*^2^ statistics in which substantial heterogeneity was defined as an *I*^2^ > 50% (34). Sensitivity analysis was conducted by removing one study at a time to re-examine the reliability and conclusiveness of the available evidence. We evaluated potential publication bias with funnel plots and Egger’s tests for an outcome reported in 10 or more studies. A *p-*value of < 0.05 was deemed statistically significant.

## 3. Results

### 3.1. Selection, characteristics, and quality of studies

**FIGURE 1:**
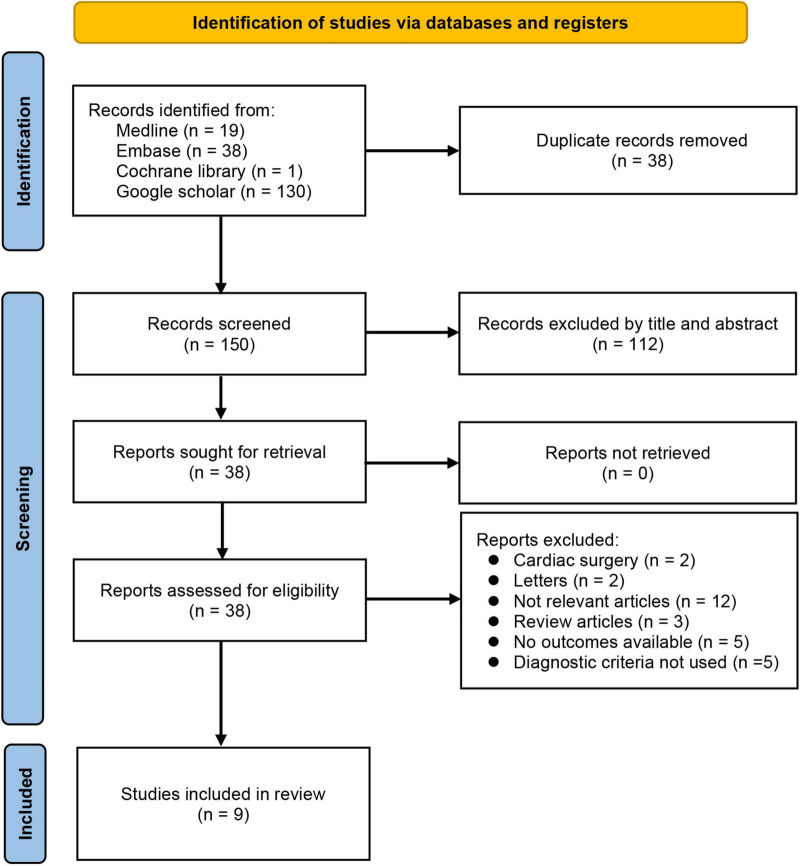
PRISMA flow diagram of study selection for the current meta-analysis.

The process of study selection for the current meta-analysis is shown in Figure 1. After deleting duplicate records and those that failed to meet our inclusion criteria from the 188 articles originally retrieved from the databases, we selected 38 potentially eligible studies for a full-text review that further excluded 29 articles (Figure 1). Finally, nine retrospective studies published between 2010 and 2021 involving 3,743 patients were included for the present meta-analysis (25, 29, 35–41).

**FIGURE 2:**
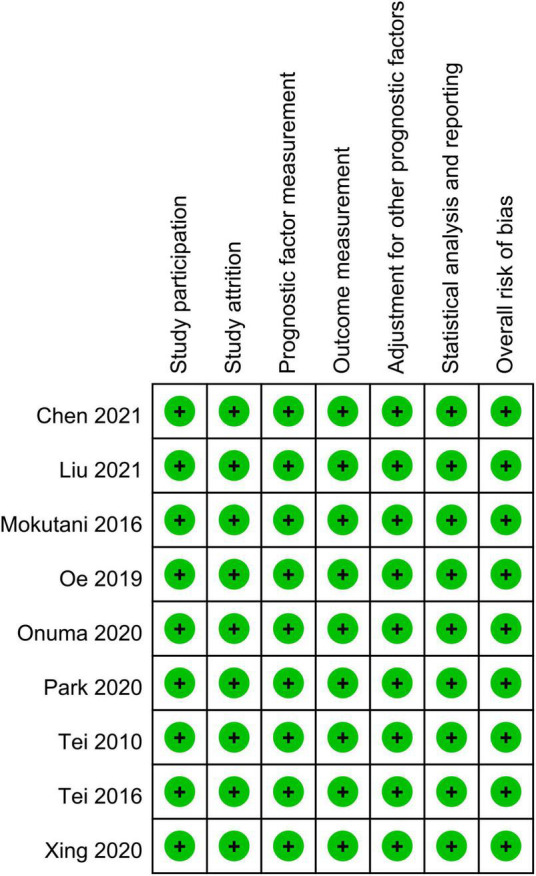
Summary of different categories of risk of bias of the included studies. Green, low risk of bias.

The characteristics of the eligible studies for the current study are shown in Table 1. The age of the participants ranged between 62 and 86 years with the proportion of males being 18.2–91.3%. Six studies provided information on BMI of the participants (25, 29, 35, 36, 38, 41) that ranged from 22 to 24 kg/m^2^, while relevant data were not given in the other three studies (37, 39, 40). All studies focused on non-cardiac surgery, including orthopedic (four studies) (25, 36, 37, 41), abdominal (three studies) (35, 39, 40), and thoracic (one study) (38) procedures. However, one study reported the enrollment of patients undergoing non-cardiac surgery without further details (29). The diagnosis of POD was according to the Confusion Assessment Method (CAM) (seven studies) (29, 35, 36, 38–41) and Diagnostic and Statistical Manual of Mental Disorders (two studies) (25, 37). The postoperative follow-up period of our included studies varied widely from 3 to 30 days. The incidence of POD ranged from 6.4 to 35% according to the included studies, which were conducted in three countries, including Japan (five studies) (35–37, 39, 40), China (three studies) (25, 29, 41), and Korea (one studies) (38). The risk of bias evaluated by the QUIPS tool (Figure 2) is shown in Figure 2. As a whole, the overall risk of bias was deemed low.

**TABLE 1:** Characteristics of studies (*n* = 9).

Studies	Age (years)^a^	Male (%)	BMI (kg/m^2^)^a^	N	Surgery	Incidence of delirium	Diagnostic criteria for delirium	Follow-up (days)	Country
Chen et al. (25)	71 vs. 66	28.7	24 vs. 24	994	Total joint arthroplasty	6.7%	DSM-V	7	China
Liu et al. (29)	70 vs. 68	58.4	23 vs. 24	361	Non-cardiac surgery	19.9%	CAM; CAM-ICU	3	China
Mokutani et al. (35)	80.2	57.1	23	156	Colorectal cancer resection	21.8%	CAM	30	Japan
Oe et al. (36)	73 vs. 62	18.2	22 vs. 23	319	Spinal surgery	9.4%	CAM	30	Japan
Onuma et al. (37)	79 vs. 79	45.8	NA	299	Spinal surgery	17.7%	DSM-V	14	Japan
Park et al. (38)	69 vs. 65	91.3	22 vs. 24	1011	Lung tumor resection	6.4%	CAM	9^‡^	Korea
Tei et al. (39)	78 vs. 75	57.4	NA	129	Colorectal cancer resection	10.9%	CAM	30	Japan
Tei et al. (40)	86 vs. 79	55.9	NA	311	Colorectal cancer resection	14.1%	CAM	30	Japan
Xing et al. (41)	74 vs. 72	42.9	23 vs. 22	163	Surgery for femur fracture	35%	CAM	7	China

DSM-V, Diagnostic and Statistical Manual of Mental Disorders, Fifth Edition; CAM, Confusion Assessment Method.

^a^Presented as PNI vs. control group; NA, not available.

^‡^Length of stay in hospital (median).

### 3.2. Results of syntheses

#### 3.2.1. Primary outcome: Association between PNI and postoperative delirium

**FIGURE 3:**
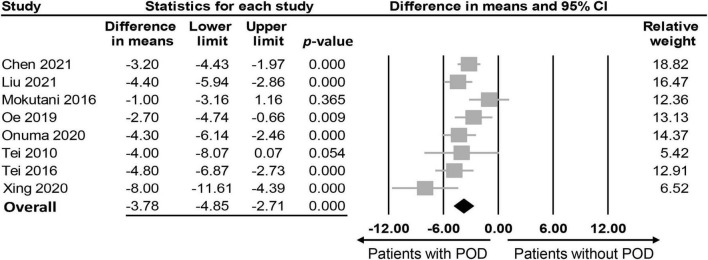
Forest plot comparing values of prognostic nutritional index (PNI) between postoperative delirium (POD) and non-POD groups, showing a lower PNI in the POD group compared to the non-POD group (MD: –3.78, 95% CI: –4.85 to –2.71, *p* < 0.0001, *I*^2^ = 54.2%). CI, confidence interval.

**FIGURE 4:**
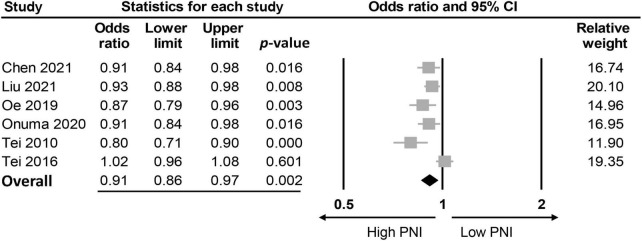
Forest plot demonstrating a negative correlation between risk of postoperative delirium (POD) and prognostic nutritional index (PNI) (OR: 0.91, 95% CI: 0.86–0.97, *p* = 0.002, *I*^2^ = 71%). CI, confidence interval.

**FIGURE 5:**
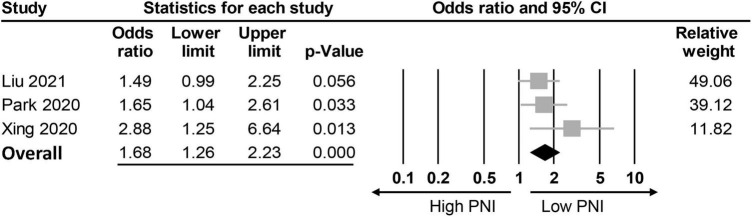
Forest plot showing a negative association between risk of postoperative delirium (POD) and prognostic nutritional index (PNI) (Cut-off values: 50) (OR: 1.68, 95% CI: 1.26–2.23, *p* < 0.0001, *I*^2^ = 0%). CI, confidence interval.

The association between PNI and the incidence of POD were assessed in three different ways among the included studies; while eight studies compared the preoperative PNI values between the POD and non-POD groups, the association of PNI with the risk of POD was investigated with the former being considered either to be a continuous parameter or a binary variable (i.e., low vs. high) in six and three studies, respectively. Eight studies provided the preoperative PNI values in the POD and non-POD groups (25, 29, 35–37, 39–41). Our meta-analysis showed lower PNI values among patients in the POD group (MD: −3.78, 95% CI: −4.85 to −2.71, *p* < 0.0001, *I*^2^ = 54.2%) compared to those in the non-POD group (Figure 3) (25, 29, 35–37, 39–41). In the six studies (25, 29, 36, 37, 39, 40) that used PNI as a continuous parameter for POD risk prediction, multivariable logistic regression was adopted as the analytical tool. Pooled results showed that a higher PNI was associated with a lower risk of POD (OR: 0.91, 95% CI: 0.86–0.97, *p* = 0.002, *I*^2^ = 71%) (Figure 4). In three studies (29, 38, 41), the PNI was treated as a binary variable [i.e., low vs. high; cut-off point: 50 in two studies (29, 38), 47.45 in one study (41)] to predict the risk of POD. The results demonstrated that a lower PNI (i.e., < 50) was correlated with a higher risk of POD (OR: 1.68, 95% CI: 1.26–2.23, *p* < 0.0001, *I*^2^ = 0%) (Figure 5). Sensitivity analysis demonstrated result consistency using the three approaches.

#### 3.2.2. Subgroup analysis

Subgroup analysis based on the type of surgery showed a negative correlation between PNI and the risk of POD in patients undergoing orthopedic surgery (Supplementary Figure 1) (25, 36, 37) but not in those undergoing abdominal surgery (Supplementary Figure 2) (39, 40).

#### 3.2.3. Association of patient characteristics and comorbidities with postoperative delirium

Individuals in the POD group were older than those in the non-POD group (MD: 1.97, 95% CI: 0.72–3.22, *p* = 0.002, eight studies) (25, 29, 35–37, 39–41), while there was no difference in BMI (MD: −0.09, 95% CI: −0.31 to 0.12, *p* = 0.39, five studies) (25, 29, 35, 36, 41) and surgical time (MD: 4.88, 95% CI: −6.3 to 16.05, *p* = 0.39, eight studies) (25, 29, 35–37, 39–41) between the two groups. In addition, health status (i.e., ASA-PS) (OR = 1.15, 95% CI: 0.85–1.55, *p* = 0.36, six studies) (25, 29, 35, 39–41), the incidence of hypertension (OR = 1.22, 95% CI: 0.91–1.64, *p* = 0.19, four studies) (25, 29, 40, 41) and diabetes mellitus (OR = 1.05, 95% CI: 0.76–1.45, *p* = 0.76, six studies) (25, 29, 37, 39–41) as well as the prevalence of male gender (OR = 0.99, 95% CI: 0.81–1.22, *p* = 0.93, eight studies) (25, 29, 35–37, 39–41) were not risk factors for POD. Sensitivity analysis of the above parameters showed consistent findings, indicating a stable result.

## 4. Discussion

Although PNI fits the criteria for an ideal risk estimation tool including non-invasiveness, easiness to perform, low cost, and being standardized (42), its role in the prediction of the risk of POD has not been addressed. The current meta-analysis of nine observational studies enrolling 3,743 adult patients undergoing non-cardiac surgery, which was the first to investigate the correlation between preoperative PNI and the risk of POD, demonstrated an association of a low PNI with an increased risk of POD. Our results demonstrated an 1.68-fold increase in the risk of POD in patients with a low PNI (i.e., cut-off value of < 50). Moreover, while our results showed that the risk of POD was age-dependent, other factors including BMI, surgical time, ASA-PS, male gender diabetes mellitus, and hypertension were not risk factors for POD.

Although the etiology of POD remains unclear, animal studies have shed light on some possible mechanisms such as neurotransmitter imbalance and neuroinflammation (43). Besides, subclinical cerebrovascular events may also contribute to an increased risk of POD (43). Despite the fact that circulating cytokine levels may not accurately reflect the degree of neuroinflammation, a recent meta-analysis demonstrated that preoperative interleukin-6 concentrations were associated with the development of POD, suggesting a relationship between a preoperative inflammation status and POD (44). Besides inflammation, malnutrition, which has been reported in 10 to 50% of older surgical patients due to decreased food intake and disease-related changes in metabolism (45–47), has also been found to contribute to the development of POD. In concert with the finding that poor nutrition is related to several known risk factors for POD, including functional and cognitive impairment, life dependency, and an increased risk of depression (48–50), other studies have identified malnutrition reflected by a low-serum albumin level as a major risk factor for POD among surgical patients (51–53). Furthermore, a recent umbrella review of systematic investigations confirmed the negative impact of a low serum albumin level on the development of POD (54), highlighting the importance of preoperative identification and correction of this modifiable risk factor.

PNI, which can be conveniently calculated from total peripheral lymphocyte count and serum albumin concentration from routine blood samples, has been reported to reflect the immune-nutritional status of patients (55). Considering the contributions of inflammation and malnutrition to the development of POD, PNI may reasonably serve as an indicator for the risk of POD. Our study is the first meta-analysis to demonstrate a negative association of PNI with the risk of POD (OR: 0.91). When a PNI cut-off value of less than 50 was used, patients with a small PNI had a 1.68-fold increased risk of POD compared to those with a relatively high PNI. In contrast to the recent popular utilization of circulating inflammation-related biomarkers (e.g., interleukin-6) for predicting the development of POD (44, 56, 57), PNI has the additional merit of including the patient’s nutritional status.

Our finding of an older age among patients in the POD group compared to those without POD was consistent with that of a previous study (58). Nevertheless, the present meta-analysis showed no association of other patient characteristics or comorbidities such as gender, BMI, and the prevalence of hypertension with the development of POD. Despite the demonstration of a negative correlation between BMI and the risk of POD in a previous study (59), we found no such association possibly due to the recruitment of patients without obesity in most of our included studies (i.e., BMI < 25 kg/m^2^ in six studies with no detail being given in three studies) (Table 1). Besides, although previous clinical studies showed that prolonged surgical/anesthetic time was a risk factor for POD (26, 60, 61), our findings did not support this relationship.

Analysis of 18 relevant meta-analyses in a recent umbrella have identified a number of consistent risk factors for POD including pre-existing cognitive impairment, increasing age, cerebrovascular disease, end stage renal failure, higher ASA score, low albumin, psychiatric disorders, and intraoperative blood transfusion (54). On the other hand, other risk factors for POD may vary with the study populations (54). For instance, the effect of gender on the risk of POD may be influenced by the type of surgery as reflected by a higher risk of POD for male patients undergoing vascular procedures (OR 1.30) compared to those receiving ENT surgery (OR 1.94) (54). However, such a gender impact was not observed in patients subjected to surgery in other disciplines (54). Our finding of a lack of correlation between POD and the reported risk factors such as gender and surgical time may be attributed to the inclusion of various surgeries. This was supported by our demonstration of a negative association between PNI and the risk of POD in patients undergoing orthopedic surgery, but not in those undergoing abdominal surgery (Supplementary Figures 1, 2). Our result was consistent with that of a recent meta-analysis that reported a potential impact of surgery type on the association between preoperative circulating inflammatory mediator levels and POD (44).

Although POD commonly occurs 2–5 days after surgery (43), there was a wide variation in follow-up period (i.e., 3–30 days) (Table 1) in our included studies. This may be attributed to the fact that some studies did not set POD as their primary outcomes, Besides, despite the setting of POD as primary outcomes in other studies, the authors did not provide specific data within postoperative 5 days. Therefore, a variation in follow-up period may bias our results. Nevertheless, the reporting of a wide time frame of POD onset (i.e., 0–14 days) in one of our included studies (25) underscored the possibility of a delayed onset of the condition. Another potential confounder that may bias our findings was the presence of depression or cognitive impairment among the participants. A previous study focusing on older individuals aged over 60 years reported a 15.5 times higher risk of depression among those with malnutrition compared to those without (62), indicating a strong correlation between malnutrition and the occurrence of depression. On the other hand, another study investigating the impact of depression on POD in patients receiving spinal surgery identified depression as an independent risk factor for POD (63). Taken together, the association of PNI with POD may be attributed to the presence of depression. Nevertheless, the fact that none of our included studies provided information about depression in their recruited subjects precluded an analysis of its impact on our study outcome.

Several limitations in the current meta-analysis need to be taken into consideration. First, because this study focused on patients receiving general anesthesia, our results may not be extrapolated to those undergoing regional anesthesia. Second, despite the use of validated measures for estimating the occurrence of POD in the present study, the heterogeneity remained high in our primary outcome. Therefore, judicious interpretation of our results is needed. Third, the retrospective design of all the included studies could not exclude the effects of potential confounders (e.g., intraoperative hemodynamic change) (61), thereby precluding the elucidation of a causal relationship between POD and PNI. Fourth, although trial sequential analysis (TSA) is essential for assessing the robustness of evidence from randomized controlled trials based on sample size, it was not designed for observational studies. As a result, TSA could not be conducted for the current study. Finally, whether the severity of delirium is related to the value of PNI remains unclear. Prospective randomized controlled trials, if possible, may help in addressing these issues.

Our results suggest that PNI may be recommended as part of routine assessment for the risk of POD, especially in older patients. Based on PNI, patients at risk of POD could be assessed for the necessity of receiving preoperative prophylactic interventions [e.g., avoidance of perioperative polypharmacy and prolonged fluid fasting as well as preoperative pain management (43)] or intraoperative measures [e.g., anesthesia depth monitoring, multimodal opioid-sparing analgesia, dexmedetomidine, and optimization of intraoperative hemodynamics (43, 64)] to minimize the risk of POD. Nevertheless, the optimal cut-off values of PNI for risk prediction of POD in different clinical settings remain to be determined. Notwithstanding evidence of a possible reduction in postoperative medical complications through preoperative nutritional interventions (65), further studies are warranted to investigate the prophylactic effectiveness of preoperative nutritional management against POD in older patients with a low PNI as well as to explore the association between PNI and delirium in the non-surgical setting.

## 5. Conclusion

Our results validated the presence of an association between preoperative PNI and the risk of POD in a variety of surgical patient populations. Patients with a PNI less than 50 may have a nearly twofold increased risk of delirium compared to those with a relatively high index. Incorporation of this simple screening predictor into clinical practice may be recommended in the older patients.

## Data availability statement

The original contributions presented in this study are included in the article/Supplementary material, further inquiries can be directed to the corresponding authors.

## Author contributions

K-CH and C-CC: conceptualization. C-WH: methodology. C-CK: software. I-WC: validation. C-NH: formal analysis and investigation. K-CH: resources, data curation, and visualization. K-CH and C-KS: writing—original draft preparation and writing—review and editing. C-KS: supervision. All authors have read and agreed to the published version of the manuscript.
