# Supplementary material for: Association of preoperative prognostic nutritional index with risk of postoperative delirium: A systematic review and meta-analysis
Source: Front Med (Lausanne). 2023 Jan 9;9:1017000. doi: 10.3389/fmed.2022.1017000 (PMC9868631; doi:10.3389/fmed.2022.1017000)
Supplement: Supplementary file 1 [file Data_Sheet_1.docx]

**Association of preoperative prognostic nutritional index with risk of postoperative delirium: A systematic review and meta-analysis**

**Supplementary Materials**

**Supplemental table 1.** Literature search strategy for MEDLINE

**Supplemental figure 1.** Forest plot demonstrating the association of prognostic nutritional index (PNI) with risk of postoperative delirium (POD) in patients undergoing orthopedic surgery.

**Supplemental figure 2.** Forest plot showing the association between prognostic nutritional index (PNI) and risk of postoperative delirium (POD) in patients undergoing abdominal surgery.

**Supplemental table 1.** Search strategies for Medline

| 1 | ("(operative or surgical) adj4 (procedure* or technique*)" or "surger*" or "operation*").mp. |
| --- | --- |
| 2 | exp "Surgical Procedures, Operative"/ |
| 3 | ("cognitive adj4 (dysfunction or decline or impairment or deficit*)" or "POD" or "postoperative cognitive dysfunction" or "mini-mental state examination " or "neuropsychological" or "MMSE " or "Cognition" or "delirium" or "postoperative delirium" or "neurocognit*" or "Confusion Assessment Method").mp. |
| 4 | exp "Postoperative Cognitive Complications"/ or exp "Delirium"/ |
| 5 | ("Prognostic Nutritional Index" or "Prognostic Nutritional Indices").mp |
| 6 | (1 or 2) and (3 or 4) and 5 |

**Supplemental figure 1.** Forest plot demonstrating the association of prognostic nutritional index (PNI) with risk of postoperative delirium (POD) (Odd ratio: 0.9, 95% confidence interval: 0.86 to 0.94, I^2^=0%) in patients undergoing orthopedic surgery.

**Supplemental figure 2.** Forest plot showing the association between prognostic nutritional index (PNI) and risk of postoperative delirium (POD) (Odd ratio: 0.91, 95% confidence interval: 0.72 to 1.15, I^2^=91.9%) in patients undergoing abdominal surgery.
